# Parliament and the Profession

**Published:** 1918-07-13

**Authors:** 


### PARLIAMENT AND THE PROFESSION.

### Army Dental Service.

MR. MACPHERSON informed Mr. Pennefather that arrangements are in progress with the object of all dental examinations in the Army being in future carried out by dental officers.

### Stafford Infirmary Hospital.

SIR WALTER ESSEX asked the Financial Secretary to the War Office whether the Stafford Infirmary is allowed by the Government 3s. 3d. a day for the maintenance of wounded soldiers, while the cost per occupied bed is 4s. 4d., and whether, in view of the straitened circumstances of the institution, he will increase the contribution. Mr. Forster said he would inquire into the matter.

### Women Doctors.

MR. FORSTER informed Sir Robert Newman that women serving as whole-time doctors in the Army for service at home and abroad receive the same pay, rations, travelling allowances, and gratuity as temporary commissioned officers of the Royal Army Medical Corps. Those serving for home duty only, on temporary engagements, are treated in the same way as civilian medical men similarly employed. All officers have their letters censored. It is not proposed to grant commissions to women doctors.

### A Curious Error.

MR. CHANCELLOR asked the President of the Local Government Board how many of the five small-pox deaths in the County of Suffolk reported in the quarterly return of the Registrar-General for the first three months of 1918 were civil, military, and naval, respectively; and what was the vaccinal condition in each case. Mr. Hayes Fisher replied that the cases referred to were wrongly entered as small-pox. They should have been entered as whooping-cough.

### Medical Treatment of School Children.

MR. HERBERT FISHER informed Mr. Rowntree that in 1917 thirty-nine local education authorities in England and Wales did not submit schemes for medical treatment of school children. In 1916 the number of such authorities was forty-three.

